# Stereotactic Body Radiation Therapy in the Management of Recurrent Unresectable Retroperitoneal Ganglioneuroma

**DOI:** 10.7759/cureus.102628

**Published:** 2026-01-30

**Authors:** Bradley Callas, Shyam Jani, Isheeta S Govardhan, Nitika Thawani, Shyamal Patel

**Affiliations:** 1 School of Medicine, Creighton University School of Medicine, Phoenix, USA; 2 Department of Radiation Oncology/Medical Physics, St. Joseph’s Hospital and Medical Center, Phoenix, USA; 3 Department of Surgery, Creighton University School of Medicine, Phoenix, USA; 4 Department of Radiation Oncology, St. Joseph’s Hospital and Medical Center, Phoenix, USA

**Keywords:** clinical case report, ganglioneuroma, neurogenic tumor, radiation oncology and clinical oncology, retroperitoneal mass, stereotactic body radiotherapy (sbrt), targeted radiation therapy, unresectable ganglioneuroma

## Abstract

Ganglioneuromas are rare, benign tumors arising from neural crest cells, typically managed with surgical resection. We present a unique case of a 35-year-old man with a history of ganglioneuroma resected in early childhood, who developed a recurrent retroperitoneal mass more than 30 years later. Imaging revealed a 12 cm lesion encasing critical structures, rendering it surgically unresectable. The patient underwent stereotactic body radiotherapy (SBRT), receiving 40 Gy in five fractions using volumetric modulated arc therapy (VMAT). Treatment was well-tolerated with no significant toxicity. Follow-up imaging over two years demonstrated stable disease without progression or symptom recurrence. To our knowledge, this is the first reported case of SBRT utilized for a retroperitoneal ganglioneuroma, highlighting its potential as a noninvasive alternative in cases where surgery is contraindicated. This case underscores the importance of long-term surveillance in patients with previously resected ganglioneuromas and suggests that SBRT may be a viable treatment option when surgical intervention poses significant risks.

## Introduction

Ganglioneuromas are rare, benign neoplasms originating from sympathetic ganglion cells, classifying them as neural crest-derived tumors. These tumors can arise anywhere along the sympathetic chain, including the posterior mediastinum and retroperitoneum [1]. However, ganglioneuromas of the adrenal glands account for approximately 50%-60% of reported cases [2].

Due to their slow growth, ganglioneuromas are often asymptomatic and discovered incidentally. When they do become symptomatic, they can cause mass effect, such as abdominal discomfort, spinal cord compression, or respiratory symptoms if the tumor is in the thorax or mediastinum [3,4]. In rare cases, these tumors may secrete catecholamines or other hormones, leading to systemic symptoms [5].

Imaging plays an important role in the diagnosis and characterization of ganglioneuromas. On computed tomography (CT), these tumors appear as well-defined, low-attenuation homogeneous masses with possible punctate calcifications [6,7]. Magnetic resonance imaging (MRI) typically reveals low signal intensity on T1-weighted images and heterogeneous high signal intensity on T2-weighted images; a characteristic whorled appearance on T2 images often appears due to interlacing bundles of Schwann cells and collagen fibers [7,8].

Given the usually benign nature of ganglioneuromas, active observation has been increasingly accepted for asymptomatic patients or those with high surgical risk [9]. Since chemotherapy has not shown to be an effective treatment option, surgical resection remains the primary and most effective treatment for ganglioneuromas [10]. Complete excision is preferable when feasible and is associated with excellent outcomes and a low risk of recurrence even in cases with residual tumor [11]. In cases where surgery is contraindicated due to tumor location, patient comorbidities, or potential morbidity, alternative therapeutic strategies are necessary. Stereotactic body radiotherapy (SBRT) is a precise, noninvasive treatment modality that delivers high-dose radiation to targeted areas with minimal exposure to surrounding tissues [12]. While SBRT has shown strong evidence for local control in neuroendocrine neoplasms and benign intracranial tumors, its use for the treatment of ganglioneuromas has not been previously described in published studies or guidelines [13].

This case study presents the first documented use of SBRT in treating a retroperitoneal ganglioneuroma, highlighting its potential as a viable alternative for patients who are not candidates for surgical intervention.

## Case presentation

A 35-year-old man with a past medical history of psoriasis and ganglioneuroma presented to the hospital with abdominal pain and intractable vomiting. He was first diagnosed with a ganglioneuroma at 18 months of age and underwent surgical resection. A recurrence at four years old necessitated a second surgery.

In February 2021, the patient developed abdominal bloating that worsened with food intake. On physical examination, his abdomen was mildly distended but non-tender. CT imaging revealed a possible partial small bowel obstruction (SBO), as well as a retroperitoneal mass measuring 8 cm. He was treated with nasogastric tube decompression for the SBO with subsequent improvement. Surgical consultation was obtained, and further imaging and biopsy were recommended.

MRI demonstrated a 12 cm soft tissue mass in the aortoiliac retroperitoneum extending from the mid-lumbar spine down to S1 with the encasement of the mid-left ureter, resulting in mild left-sided hydroureteronephrosis. CT-guided biopsy by interventional radiology confirmed the diagnosis of ganglioneuroma. SBO was determined to be secondary to the ganglioneuroma, and treatment was recommended to prevent the further growth of the lesion and the redevelopment of SBO.

The patient was referred to outpatient oncology, where his case was reviewed at a multidisciplinary tumor board including radiology, urology, general surgery, radiation oncology, pathology, and medical oncology. Urology and general surgery deemed the mass surgically unresectable due to its location and involvement with critical structures. The recommendation for definitive radiation therapy was made, given the locally aggressive nature of the lesion and symptomatic burden. Specifically, SBRT was recommended to allow for dose escalation and offer a higher likelihood of stopping further lesion growth.

**Figure 1:**
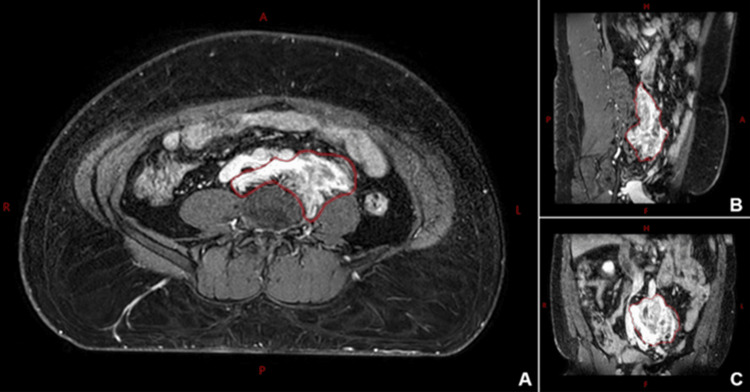
Retroperitoneal ganglioneuroma mass on a post-contrast T1-weighted MRI in axial (A), sagittal (B), and coronal (C) views. The drawn gross tumor volume (GTV) is shown in red. MRI: magnetic resonance imaging

The patient was simulated on a Brilliance Big Bore CT scanner (Philips, Amsterdam, Netherlands) in a head-first supine position with BodyFix immobilization (Elekta, Stockholm, Sweden). Non-contrast and contrast-enhanced CT scans were acquired at 1.5 mm slice thickness. Diagnostic T2-weighted and post-contrast T1-weighted magnetic resonance (MR) images on an Ingenia 1.5T scanner (Philips, Amsterdam, Netherlands) were acquired and registered with the planning CT to assist with gross tumor volume (GTV) delineation (Figure 1). The final planning target volume (PTV) was a 5 mm uniform expansion of the GTV.

**Figure 2:**
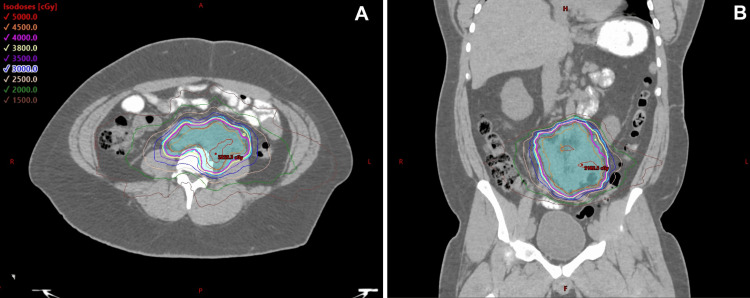
Isodose distributions in the axial (A) and coronal (B) planes of the CT planning dataset with a prescription of 40 Gy in five fractions. Anterior and lateral borders of the PTV (cyan) are undercovered to spare the small bowel and ureter. CT, computed tomography; PTV, planning target volume

A treatment plan was developed using the Eclipse treatment planning system (version 15.6; Varian Medical Systems, Palo Alto, CA) and Acuros XB version 15.6 as the dose calculation algorithm. A volumetric modulated arc therapy (VMAT) technique with three full arcs was designed to deliver a total of 40 Gy in five fractions to the PTV. Dose constraints for critical structures were evaluated using the Timmerman normal tissue dose-volume tables [14]. The anterior and lateral PTV borders were underdosed to meet small bowel and ureter constraints. The final prescription dose coverage of the PTV and GTV was 85.2% and 97.5%, respectively; the 95% dose coverage of the PTV and GTV was 93.6% and 99.5%, respectively (Figure 2). Cone beam CT imaging was used for pre-treatment setup verification prior to treatment delivery on a Varian TrueBeam (Varian Medical Systems, Palo Alto, CA) linear accelerator for each fraction.

The patient completed SBRT in early April 2021 and tolerated treatment well without significant toxicity. Due to the mass’s proximity to the ureters, he was referred to urology for the evaluation of possible ureteral stent placement. Renal ultrasound showed only mild left hydronephrosis with preserved bilateral ureteral jets. A mercaptoacetyltriglycine (MAG3) Lasix renal scan showed symmetric function with normal washout and no evidence of obstruction. In light of these findings and the absence of symptoms, urology recommended observation without stent placement.

Follow-up MR and ultrasound imaging in December 2022 and July 2023, respectively, showed a stable tumor size without symptoms. Last clinical follow-up in July 2025 revealed him to be without any clinical signs of recurrence. The patient declined repeat imaging at that time due to cost limitations.

## Discussion

Ganglioneuromas are benign tumors originating from neural crest cells, typically exhibiting indolent behavior. However, their recurrence in adulthood remains poorly understood. Proposed mechanisms for tumor dormancy and late recurrence include cellular quiescence, immune surveillance, and limited angiogenesis [15]. These factors may prevent tumor proliferation until changes in the microenvironment permit reactivation. Some studies have suggested that late metastases may result from early disseminated tumor cells constrained by immunosurveillance, supporting the concept of tumor dormancy in ganglioneuromas [15,16].

CT and MR imaging play an important role in diagnosis and determining treatment options due to well-defined tumor characteristics, as well as identifying proximity to adjoining structures and nerves [6-8]. Surgical resection is the primary treatment for ganglioneuromas and is often curative. However, a complete resection may be precluded due to irregular borders or the invasion of nearby vasculature or organs [16]. Incomplete surgical resection can still be an effective treatment option due to the low risk of recurrence and can be considered in cases of stable ganglioneuromas [17]. For recurrent tumors, aggressive surgery can be pursued with the risk of additional complications [18]. Recent research has identified activated protein kinase B (AKT) as a potential driver in ganglioneuroma tumorigenesis, leading to the exploration of mammalian target of rapamycin (mTOR) inhibitors as a means to reduce tumor size preoperatively and potentially decrease surgical morbidity [19].

There are multiple cited cases in the literature of radiotherapy for neurogenic tumors [20-22]. Rakici et al. treated a recurrent presacral ganglioneuroma using a simultaneous integrated boost (SIB) approach with 56 Gy and 50.4 Gy in 28 fractions to the defined GTV and clinical target volume (CTV) [23]. The presented case in this study mirrored the findings of postsurgical progression and posed surgical challenges due to vessel and ureter involvement. The specific choice of SBRT fractionation lay in the historical efficacy of treating inoperable or recurrent benign neurogenic tumors with high local control and symptom relief [24-27]. SBRT offers a noninvasive, targeted approach that has become increasingly feasible for treating tumors near critical structures due to advances in radiation delivery technology [6]. Delivery techniques such as VMAT facilitate highly conformal dose distributions with sharp dose gradients outside the target volume, allowing ablative doses to be delivered directly to the tumor while sparing adjacent healthy tissue. Modern treatment delivery technology can leverage high-resolution imaging and daily image guidance to align and irradiate targets with high precision.

In this case, the lesion’s proximity to major vasculature limited the feasibility of another surgical resection. SBRT was chosen to enable dose escalation with the goal of achieving durable local control and inhibiting further tumor progression, despite the tumor’s relatively large size. The successful treatment of this retroperitoneal ganglioneuroma with local disease control posttreatment suggests SBRT as a promising nonsurgical alternative for controlling benign but potentially aggressive lesions when anatomical constraints prevent safe resection.

## Conclusions

This case highlights a rare instance of recurrent retroperitoneal ganglioneuroma occurring more than 30 years after initial resection. Due to the tumor’s location and its proximity to critical structures, surgical resection carried significant morbidity. SBRT was pursued as a noninvasive alternative due to its demonstrated efficacy and local control in other benign neurogenic tumors. The treatment was well-tolerated and led to local disease control with no progression at follow-up.

To our knowledge, this is the first reported case of SBRT used for a retroperitoneal ganglioneuroma. It underscores the importance of long-term surveillance in patients with resected ganglioneuromas and a multidisciplinary approach to aid in patient care management. SBRT may be a viable treatment option when surgery is contraindicated or considered high-risk.
